# Supplementary material for: Pool PaRTI: A PageRank-Based Pooling Method for Identifying Critical Residues and Enhancing Protein Sequence Representations
Source: bioRxiv. 2025 Mar 17:2024.10.04.616701. Preprint. [Version 2] doi: 10.1101/2024.10.04.616701 (PMC11956911; doi:10.1101/2024.10.04.616701)
Supplement: 1 [file NIHPP2024.10.04.616701V2-supplement-1.pdf]

bioRxiv preprint doi: https://doi.org/10.1101/2024.10.04.616701; this version posted March 17, 2025. The copyright holder for this preprint (which was not certified by peer review) is the author/funder, who has granted bioRxiv a license to display the preprint in perpetuity. It is made available under aCC-BY 4.0 International license.

A Appendix / Supplemental Material

A.1 Theoretical expectation and standard deviation of Jaccard index under random weight assignment

In this section, we derive the expectation and standard deviation of the Jaccard index when two sets of indices are selected randomly from a total of N elements.

A.1.1 Expectation of the Jaccard Index

Let $p = k/N$ be the fraction of selected elements, where k is the number of selected elements from a total of N. The Jaccard index for two randomly selected sets of size k is given by:

$$J = \frac{|A \cap B|}{|A \cup B|}$$

where A and B are the two selected sets. The key quantities to compute are:

- **Expected size of the intersection:** Since each element is included in a set with probability p, the probability that a given element appears in both sets is p^2. Since there are N elements, the expected intersection size is:
$$\mathbb{E}[|A \cap B|] = Np^2.$$
- **Expected size of the union:** Using the inclusion-exclusion principle, the expected union size is:
$$\mathbb{E}[|A \cup B|] = 2k - \mathbb{E}[|A \cap B|] = 2Np - Np^2.$$

Thus, the expectation of the Jaccard index is:

$$\mathbb{E}[J] = \frac{\mathbb{E}[|A \cap B|]}{\mathbb{E}[|A \cup B|]} = \frac{Np^2}{2Np - Np^2}.$$

Simplifying,

$$\mathbb{E}[J] = \frac{p}{2-p}.$$

This is the expected Jaccard index for randomly sampled sets as a function of p, independent of N.

A.1.2 Variance and Standard Deviation of the Jaccard Index

To compute the variance of J, we use the formula:

$$\text{Var}(J) = \mathbb{E}[J^2] - (\mathbb{E}[J])^2.$$

bioRxiv preprint doi: https://doi.org/10.1101/2024.10.04.616701; this version posted March 17, 2025. The copyright holder for this preprint (which was not certified by peer review) is the author/funder, who has granted bioRxiv a license to display the preprint in perpetuity. It is made available under aCC-BY 4.0 International license.

Following standard combinatorial derivations from random set theory, the variance of J is:

$$\mathrm{Var}(J) = \frac{p(1-p)}{(2-p)^2(N-1)}.$$

Taking the square root, the standard deviation of J is:

$$\mathrm{SD}(J) = \sqrt{\frac{p(1-p)}{(2-p)^2(N-1)}}.$$

A.2 The choice of metrics

For evaluation of the performance gains that the Pool PaRTI embeddings yield, we use the Matthews Correlation Coefficient (MCC) and Area Under the Precision-Recall Curve (AUPRC), chosen for their robustness in assessing binary classification performance across unbalanced datasets, alongside accuracy. For multi-label prediction, we also report the Jaccard index to evaluate performances. For single label and multilabel binary classifications, we employed different metrics to assess model predictive performances under the influence of the input embeddings resulting from alternative pooling methods.

A.2.1 Precision @10%

Precision at 10% (P@10%) is a useful metric for ranking-based tasks, measuring how many of the top 10% of predictions are correct. In this context, 10% refers to the 10% of the size of each category in balanced subsampling. For example, if we sample a balanced subset from an imbalanced dataset such that each category has 30 representatives, we report the precision for the labels of the top 3 proteins that have the highest cosine similarity to the query protein, based on the label of the query protein. This is particularly relevant for applications where the model needs to rank the most relevant items higher. It is defined as:

$$\text{Precision@10\%} = \frac{\text{Correct Predictions in top 10\%}}{\text{Total Predictions in top 10\%}} \tag{1}$$

This metric helps evaluate the effectiveness of embeddings in ranking scenarios and ensures that highly relevant items are correctly prioritized [26].

A.2.2 Mean Reciprocal Rank (MRR)

The Mean Reciprocal Rank (MRR) is a ranking evaluation metric that computes the average of the reciprocal ranks of the first correct prediction. It is particularly useful in search systems, where finding the first relevant result is critical. MRR is computed as follows:

bioRxiv preprint doi: https://doi.org/10.1101/2024.10.04.616701; this version posted March 17, 2025. The copyright holder for this preprint (which was not certified by peer review) is the author/funder, who has granted bioRxiv a license to display the preprint in perpetuity. It is made available under aCC-BY 4.0 International license.

$$\text{MRR} = \frac{1}{|Q|} \sum_{i=1}^{|Q|} \frac{1}{\text{rank}_i} \tag{2}$$

where is the number of queries and is the rank position of the first relevant item for the -th query.

Higher MRR values indicate better performance in ranking tasks, as relevant predictions appear earlier in the ranked list [26].

A.2.3 Jaccard Index

The Jaccard Index, also known as Jaccard similarity, is a measure used to compare the similarity between two sets. It is particularly useful in multi-label classification problems, where it measures the overlap between predicted and true label sets. It is defined as:

$$\text{Jaccard Index} = \frac{|Y_{true} \cap Y_{pred}|}{|Y_{true} \cup Y_{pred}|} \tag{3}$$

where is the set of true labels and is the set of predicted labels.

The Jaccard Index ranges from 0 to 1, with higher values indicating greater similarity between the predicted and actual labels. It is particularly effective in evaluating model performance in multi-label scenarios, ensuring that both precision and recall are considered simultaneously [26].

A.2.4 Accuracy

Accuracy is the simplest and most commonly used metric for evaluating classification models. It is defined as the ratio of correctly predicted instances to the total instances. Mathematically, accuracy is expressed as:

$$\text{Accuracy} = \frac{TP + TN}{TP + TN + FP + FN} \tag{4}$$

While accuracy is intuitive and easy to understand, it can be misleading for imbalanced datasets, as it does not differentiate between the types of errors (false positives and false negatives).

A.2.5 Balanced Accuracy for Multicategory Predictions

Balanced accuracy is an extension of accuracy that accounts for class imbalance in multi-category classification problems. Unlike standard accuracy, which can be skewed by dominant classes, balanced accuracy ensures that each class contributes equally to the overall performance. It is computed as the average recall across all classes:

$$\text{Balanced Accuracy} = \frac{1}{C} \sum_{i=1}^{C} \frac{TP_i}{TP_i + FN_i} \tag{5}$$

bioRxiv preprint doi: https://doi.org/10.1101/2024.10.04.616701; this version posted March 17, 2025. The copyright holder for this preprint (which was not certified by peer review) is the author/funder, who has granted bioRxiv a license to display the preprint in perpetuity. It is made available under aCC-BY 4.0 International license.

where C is the total number of classes, and TP_i and FN_i represent the true positives and false negatives for class i, respectively.

Balanced accuracy is particularly useful in scenarios where some categories are underrepresented, preventing models from favoring majority classes. In a balanced dataset, balanced accuracy is equal to the standard definition of accuracy. It provides a more fair assessment of performance across all classes and is widely used in multi-class classification tasks [27].

A.2.6 Matthews Correlation Coefficient (MCC)

The Matthews Correlation Coefficient (MCC) is a measure of the quality of binary (two-class) classifications. It takes into account true and false positives and negatives and is generally regarded as a balanced measure which can be used even if the classes are of very different sizes. The MCC is defined as:

$$\text{MCC} = \frac{TP \times TN - FP \times FN}{\sqrt{(TP + FP)(TP + FN)(TN + FP)(TN + FN)}} \tag{6}$$

where:

- TP = True Positives
- TN = True Negatives
- FP = False Positives
- FN = False Negatives

MCC returns a value between -1 and 1. A coefficient of 1 represents a perfect prediction, 0 represents a random prediction, and -1 indicates total disagreement between prediction and observation [28].

A.2.7 Area Under the Precision-Recall Curve (AUPRC)

The Area Under the Precision-Recall Curve (AUPRC) is a performance measurement for classification problems at various threshold settings, especially useful for imbalanced datasets. The Precision-Recall curve plots precision (positive predictive value) against recall (sensitivity) for different threshold values. Precision and recall are defined as:

$$\text{Precision} = \frac{TP}{TP + FP} \tag{7}$$

$$\text{Recall} = \frac{TP}{TP + FN} \tag{8}$$

The AUPRC provides a single scalar value to summarize the curve, with higher values indicating better performance. It is particularly useful when the positive class is rare, as it focuses on the performance of the positive class [29].

bioRxiv preprint doi: https://doi.org/10.1101/2024.10.04.616701; this version posted March 17, 2025. The copyright holder for this preprint (which was not certified by peer review) is the author/funder, who has granted bioRxiv a license to display the preprint in perpetuity. It is made available under aCC-BY 4.0 International license.

These metrics together provide a comprehensive evaluation framework for our models, allowing us to capture different aspects of performance, particularly in the presence of class imbalance and multi-label scenarios.

A.3 Empirical analysis of Pool PaRTI runtime scaling

To formally assess the empirical scaling of runtime for the Pool PaRTI algorithm, we executed the algorithm on 25 protein sequences, repeating each run 15 times. We then applied a logarithmic transformation to both the runtime data and the corresponding sequence lengths. A linear regression model was fitted to the log-transformed data, yielding a slope of 2.068, which aligns with our theoretical reasoning in Section 2. This slope indicates the exponent in the power-law relationship between the algorithm's runtime and the input size, providing a quantitative measure of the algorithm's scaling behavior with respect to input size.

A.4 Deep Learning Models and Configurations

A.4.1 Subcellular Localization Prediction Task

Description of the model

- 2 x (linear layer + leaky ReLU + dropout)
- Linear layer
- Input residual connection
- Sigmoid

Fixed configurations

- Initial learning rate: 0.01
- Max number of epochs: 1000
- Early stopping patience epochs: 50
- Optimizer: AdamW
- Learning rate scheduler: ReduceLROnPlateau
- Optimizer learning rate patience epochs: 10
- Learning rate reduction ratio: 0.1
- Weight Initialization: Xavier normal
- Random seed: 42
- Gradient clip value: 5.0
- batch size: 64

Hyperparameter optimization space

- Weight decay: [0.01, 0.1, 0.2]

bioRxiv preprint doi: https://doi.org/10.1101/2024.10.04.616701; this version posted March 17, 2025. The copyright holder for this preprint (which was not certified by peer review) is the author/funder, who has granted bioRxiv a license to display the preprint in perpetuity. It is made available under aCC-BY 4.0 International license.

- Dropout rate: [0.15, 0.25]
- Slope of Leaky ReLU: [0.01, 0.1]
- Exponent for imbalance penalty taming: [0.75, 1, 1.25]

A.4.2 Protein-Protein Interaction Prediction Task

Description of the model

- 1 x (linear layer + batch norm + leaky ReLU + dropout)
- 1 x (linear layer + linearly transformed residual connection + leaky ReLU)
- Linear layer

Fixed configurations

- Hidden dimensions: 1024
- Max number of epochs: 40
- Early stopping patience: 9
- Optimizer: AdamW
- Learning rate scheduler: ReduceLROnPlateau
- Optimizer learning rate patience epochs: 4
- Random seed: 42
- Weight initialization: Kaiming normal
- Gradient clip max value: 2.0
- batch size: 32

Hyperparameter optimization space

- Initial learning rate: [0.001, 0.01]
- Weight decay: [0.01, 0.1, 0.2]
- Dropout: [0.05, 0.15, 0.25]
- Reduction in learning rate ratio: [0.2, 0.5]
- Leaky ReLU slope: [0.01, 0.1, 0.2]

A.5 Compute resources for experiments

We ran all computational experiments and pooling algorithms on Tesla V100-SXM2-16GB GPUs housed in the internal Sherlock cluster. For each task, the hyperparameter optimization experiments were limited to two days on GPU. On the same GPUs, we conducted unpublished preliminary experiments in developing the Pool

bioRxiv preprint doi: https://doi.org/10.1101/2024.10.04.616701; this version posted March 17, 2025. The copyright holder for this preprint (which was not certified by peer review) is the author/funder, who has granted bioRxiv a license to display the preprint in perpetuity. It is made available under aCC-BY 4.0 International license.

PaRTI algorithm. We generated the precomputed ESM2 token embeddings on NVIDIA A100-PCIE-40GB GPUs, also housed in the internal Sherlock cluster. Token embedding generation took 70 GPU hours through the ESM2 650M model and 50 hours through protBERT model. We have computed token embeddings once for each sequence and performed several different pooling operations on the precomputed token embeddings before feeding the sequence embeddings as inputs to the respective models.
